# Associations between psychiatric comorbidity and intensive care unit length of stay, hospital length of stay, and in‐hospital mortality: A retrospective observational study

**DOI:** 10.1002/pcn5.70439

**Published:** 2026-11-02

**Authors:** Toshinori Nakamura, Takumi Momma, Tomonari Yoshizawa, Yusuke Arai, Daimei Sasayama, Atsuyoshi Mita, Hiroshi Imamura, Ryota Hashimoto, Shinsuke Washizuka

**Affiliations:** ^1^ Department of Psychiatry Shinshu University School of Medicine Matsumoto Nagano Japan; ^2^ Department of Community Mental Health Shinshu University School of Medicine Matsumoto Nagano Japan; ^3^ Division of Gastroenterological, Hepato‐Biliary‐Pancreatic, Transplantation and Pediatric Surgery, Department of Surgery Shinshu University School of Medicine Matsumoto Nagano Japan; ^4^ Department of Emergency and Critical Care Medicine Shinshu University School of Medicine Matsumoto Nagano Japan; ^5^ Department of Pathology of Mental Diseases National Institute of Mental Health, National Center of Neurology and Psychiatry Kodaira Tokyo Japan

**Keywords:** comorbidity, in‐hospital mortality, intensive care unit, length of stay, psychiatric disorders

## Abstract

**Aim:**

Psychiatric comorbidities are common among patients admitted to the intensive care unit (ICU), but findings regarding their impact on clinical outcomes remain inconsistent. Additionally, existing studies have often included patients admitted for suicide attempts or self‐harm, leaving insufficient evidence about the independent effects of psychiatric comorbidity on clinical outcomes. To address this gap, this study investigated the independent association of psychiatric comorbidity with ICU length of stay, hospital length of stay, and in‐hospital mortality among patients admitted to the ICU, excluding those admitted for suicide attempts or self‐harm.

**Methods:**

Of the 2599 patients admitted to the ICU of a university hospital, 2192 were included in the analysis. After adjusting for age, sex, body mass index, admission category, and severity score, generalized linear models were used to evaluate ICU and hospital length of stay, and logistic regression analysis was used to assess in‐hospital mortality.

**Results:**

Among the 2192 patients included in the analysis, 75 had psychiatric comorbidity. In adjusted analyses, psychiatric comorbidity was not significantly associated with ICU length of stay, hospital length of stay, or in‐hospital mortality.

**Conclusion:**

No participants were admitted to the ICU for suicide attempts or self‐harm, and psychiatric comorbidity was not an independent predictor of ICU length of stay, hospital length of stay, or in‐hospital mortality. Thus, in this ICU population, no statistically significant association was detected between psychiatric comorbidity and short‐term clinical outcomes. Therefore, caution is needed when estimating ICU outcomes based solely on the presence of psychiatric comorbidity.

## INTRODUCTION

Some patients admitted to the intensive care unit (ICU) have psychiatric comorbidities, including depression, bipolar disorder, schizophrenia, and anxiety disorders. A recent large multicenter study reported that 8.1% of ICU patients had major psychiatric comorbidities,^1^ indicating that psychiatric comorbidity is a common background factor in ICU practice.^2^

Several studies have examined the association between psychiatric comorbidity and ICU and hospital length of stay, or longer hospital stay among patients with psychiatric disorders.^3^ However, many of these analyses included patients admitted following self‐harm, such as suicide attempts or intentional drug overdose, making it difficult to evaluate the independent effect of psychiatric comorbidity itself. In addition, studies involving trauma patients or general hospitalized patients have reported an association between psychiatric comorbidity and prolonged hospital stay,^4^, ^5^, ^6^ but studies limited to ICU patients that evaluate ICU and hospital length of stay separately remain scarce.

Findings regarding mortality have also been inconsistent. A study using administrative data reported lower mortality in univariate analysis, whereas multivariable adjustment showed an increased risk of in‐hospital death among patients with psychiatric comorbidity,^7^ suggesting that conclusions may vary depending on the analytical methods and covariates used. Furthermore, among studies of ICU patients, some have reported higher ICU mortality in patients with psychiatric comorbidity,^8^ whereas others have found no significant difference.^2^, ^3^ In many previous studies, the analyzed population included patients transported after suicide attempts or self‐harm.^2^, ^3^ Although such patients are more likely to require ICU admission, they do not necessarily have a higher mortality rate. Therefore, differences in study populations may be one reason for the inconsistent findings on mortality among ICU patients with psychiatric comorbidity.

Previous studies have suggested that psychiatric comorbidities may be associated with prolonged stays in the ICU as well as in the hospital. However, these findings have been inconsistent, and differences in patient populations and study designs may limit their generalizability. In particular, evidence remains limited regarding ICU populations that do not include admissions related to suicide attempts or self‐harm. Additionally, although several studies have examined the association between psychiatric comorbidity and ICU outcomes, evidence concerning Japanese ICU populations remains scant. Therefore, this study examined the association of psychiatric comorbidity with ICU length of stay, hospital length of stay, and in‐hospital mortality. We focused on patients admitted to the ICU for physical illness, excluding cases related to suicide attempts or self‐harm, to clarify the independent impact of psychiatric comorbidity on acute clinical outcomes.

## METHODS

All patients admitted to the ICU of Shinshu University Hospital between April 2019 and March 2022 were eligible for inclusion in the study. During the study period, 2599 patients were admitted to the ICU. The exclusion criteria were age <18 years, second or subsequent ICU admission during the study period, and cases deemed inappropriate for analysis by the investigators owing to insufficient medical records. Totally, 407 patients were excluded: 139 patients <18 years, 264 with second or subsequent ICU admissions during the study period, three patients whose records could not be reviewed because access was restricted under the institutional privacy protection system, and one patient who had been admitted to the ICU despite being included in the ICU database in error. Ultimately, 2192 patients were included in the analysis (Figure 1). None of the patients were admitted to the ICU primarily because of suicide attempts or self‐harm. This reflects the clinical practice at the study site, that is, where patients presented after suicide attempts or self‐harm are generally managed in the emergency ward both initially and during the perioperative period, rather than being directly admitted to the ICU. For example, patients with severe trauma related to suicide attempts, such as pelvic fractures caused by jumping from a height, are initially managed in the emergency ward, and after surgery, patients typically return to the emergency ward rather than the ICU.

**Figure 1:**
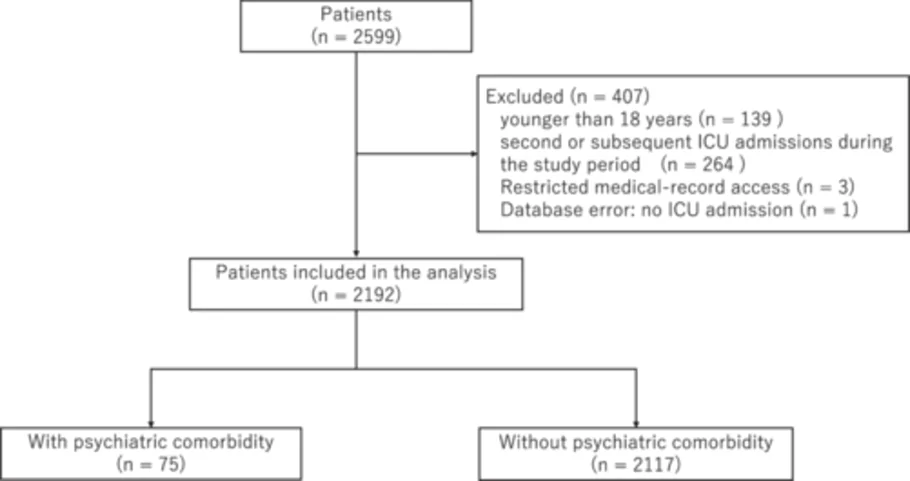
Flow diagram of the study participants. ICU, intensive care unit.

For the 2192 patients included in the analysis, the following variables were collected from the medical records: age at ICU admission; sex; body mass index (BMI); ICU length of stay (hours); hospital length of stay (days); severity at ICU admission (assessed using the Acute Physiology and Chronic Health Evaluation II [APACHE II],^9^ Acute Physiology and Chronic Health Evaluation III [APACHE III],^10^ Simplified Acute Physiology Score II [SAPS II],^11^ and Sequential Organ Failure Assessment [SOFA])^12^; ICU mortality; in‐hospital mortality; ICU admission category (non‐surgical, elective surgery, or emergency surgery); and primary diagnosis category (cardiovascular, neurological, respiratory, gastrointestinal, or other).

Psychiatric comorbidity was defined as documented ongoing treatment before the index hospitalization for disorders classified as F2, F3, or F4 according to the ICD‐10, based on a review of the medical records. Among nine previous studies that evaluated psychiatric comorbidity in ICU and related acute‐care populations,^1^, ^2^, ^3^, ^4^, ^5^, ^6^, ^7^, ^8^, ^13^ seven primarily focused on psychotic, mood/affective, and anxiety‐ and stress‐related disorders, which fall within or broadly correspond to ICD‐10 categories F2–F4.^1^, ^2^, ^3^, ^4^, ^7^, ^8^, ^13^ Based on this predominance in the previous literature, we selected F2–F4 as the diagnostic scope of psychiatric comorbidity in the present study. We included all disorders within these categories rather than selecting individual diagnoses to apply a consistent category‐based definition. Psychiatric care or consultation during ICU stay and the use of psychotropic medications before hospitalization were also assessed. For the purposes of this study, psychotropic medications were operationally defined as antipsychotics, antidepressants, mood stabilizers, antiepileptic drugs, anticholinergic drugs, anxiolytics and hypnotics, antidementia drugs, medications for attention‐deficit/hyperactivity disorder (ADHD), and medications for alcohol dependence.

This study was approved by the Medical Ethics Committee of Shinshu University School of Medicine (Study No. 5871). Because of its retrospective observational design, the requirement for written informed consent was waived. Nonetheless, information about the study, including the purpose, study period, eligible participants, use of medical records, and protection of personal information, was disclosed on the institutional website. Participants were also informed of their right to opt out from participation without any disadvantages.

Patient characteristics and clinical outcomes, that is, ICU length of stay, hospital length of stay, and in‐hospital mortality, were compared according to psychiatric comorbidity status. After examining the distributions of continuous variables, between‐group comparisons were performed using the Mann–Whitney *U* test, as the distributions were not normal. Categorical variables were compared using the chi‐square test or Fisher's exact test, as appropriate. The outcomes of interest were ICU length of stay, hospital length of stay, and in‐hospital mortality. For ICU length of stay and hospital length of stay, multivariable generalized linear models with a gamma distribution and log link were constructed, following the approach described by Austin et al.^14^ Results are presented as exponentiated regression coefficients (Exp[*β*]) with 95% confidence intervals (CIs). In‐hospital mortality was evaluated as a binary outcome using multivariable logistic regression analysis, and adjusted odds ratios (aORs) with 95% CIs were calculated. In all multivariable models, the presence or absence of psychiatric comorbidity was entered as the primary independent variable, and age, sex, ICU admission category, BMI, and severity index were included as covariates. High multicollinearity was observed among the severity indices APACHE II, APACHE III, SAPS II, and SOFA (Supporting Information S1: Table 1). Therefore, APACHE III was selected as the representative severity index based on its clinical relevance and statistical stability. No multicollinearity was observed in the model that included only APACHE III scores (Supporting Information S2: Table 2). There were no missing data for the outcomes or covariates included in the final multivariable models; therefore, all 2192 patients were included in each multivariable analysis. ICU mortality was excluded from the analysis, as no deaths occurred in the psychiatric comorbidity group. All tests were two‐sided, and statistical significance was set at *p* < 0.05. All statistical analyses were performed using spss version 30.0 (IBM Corp.).

## RESULTS

Among the 2192 patients included in the analysis, 75 (3.4%) had psychiatric comorbidity, and 2117 (96.6%) did not have any psychiatric comorbidity (Figure 1). Among the 75 patients with psychiatric comorbidity, 12, 45, and 18 had disorders classified under ICD‐10 categories F2, F3, and F4, respectively. Compared with patients without psychiatric comorbidity, those with psychiatric comorbidity were significantly younger at ICU admission, were more likely to be female, and had a significantly higher BMI. APACHE II, APACHE III, and SAPS II scores were significantly lower in patients with psychiatric comorbidity. The ICU admission category differed significantly between the two groups. Psychiatric consultations and psychotropic medication were significantly more frequent among patients with psychiatric comorbidity. Effect sizes were small for all statistically significant between‐group differences (Table 1).

**Table 1:** Comparison of intensive care unit (ICU) patients with and without psychiatric comorbidity.

Variable	With psychiatric comorbidity (*n* = 75)	Without psychiatric comorbidity (*n* = 2117)	Overall (*n* = 2192)	*p* value	Effect size
*Baseline characteristics*					
Age at ICU admission	62.0 [45.0–74.0]	70.0 [57.0–77.0]	70.0 [56.0–77.0]	<0.001*	*r* = 0.071
Sex (male)	32 (42.7%)	1244 (58.8%)	1276 (58.2%)	0.005*	Φ = 0.059
BMI (kg/m^2^)	24.0 [21.1–28.1]	22.9 [20.4–25.6]	22.9 [20.4–25.7]	0.035*	*r* = 0.045
ICU admission category				0.041*	*V* = 0.054
Non‐surgical	17 (22.7%)	269 (12.7%)	286 (13.0%)		
Elective surgery	48 (64.0%)	1550 (73.2%)	1598 (72.9%)		
Emergency surgery	10 (13.3%)	298 (14.1%)	308 (14.1%)		
Primary diagnosis category				0.562	*V* = 0.037
Cardiovascular	28 (37.3%)	973 (46.0%)	1001 (45.7%)		
Neurological	24 (32.0%)	615 (29.1%)	639 (29.2%)		
Respiratory	7 (9.3%)	201 (9.5%)	208 (9.5%)		
Gastrointestinal	7 (9.3%)	145 (6.8%)	152 (6.9%)		
Other	9 (12.0%)	183 (8.6%)	192 (8.8%)		
Severity at ICU admission					
APACHE II	18.0 [11.0–29.0]	19.0 [13.0–30.0]	19.0 [13.0–30.0]	0.038*	*r* = 0.044
APACHE III	50.0 [34.0–65.0]	55.0 [41.0–71.0]	55.0 [41.0–71.0]	0.013*	*r* = 0.053
SAPS II	32.0 [16.0–49.0]	35.0 [20.0–55.0]	34.0 [20.0–55.0]	0.039*	*r* = 0.044
SOFA	6.0 [3.0–9.0]	7.0 [4.0–9.0]	6.0 [4.0–9.0]	0.162	*r* = 0.030
*Clinical outcomes*					
ICU length of stay (h)	22.6 [18.1–73.3]	22.6 [18.8–66.9]	22.6 [18.8–67.3]	0.878	*r* = 0.003
Hospital length of stay (days)	25.0 [14.0–39.0]	23.0 [16.0–41.0]	23.0 [16.0–41.0]	0.463	*r* = 0.016
ICU mortality, *n* (%)	0 (0.0%)	63 (3.0%)	63 (2.9%)	0.275	Φ = 0.032
In‐hospital mortality, *n* (%)	2 (2.7%)	103 (4.9%)	105 (4.8%)	0.581	Φ = 0.019
Psychiatric consultation	29 (38.7%)	155 (7.3%)	184 (8.4%)	<0.001*	Φ = 0.205
Psychotropic medication use	62 (82.7%)	408 (19.3%)	470 (21.4%)	<0.001*	Φ = 0.281

*Note*: Patients admitted to the ICU with psychiatric comorbidity were younger at ICU admission, had a higher BMI, and had significantly lower APACHE II, APACHE III, and SAPS II scores than those without psychiatric comorbidity. In addition, the proportion of women was higher, the ICU admission category differed significantly, and psychiatric consultations and psychotropic medication use were significantly more frequent among patients with psychiatric comorbidity. In contrast, no significant differences were observed in ICU length of stay, hospital length of stay, ICU mortality, or in‐hospital mortality. Continuous variables are presented as median (interquartile range [IQR]). *p* values were calculated using the Mann–Whitney *U* test for continuous variables and the chi‐square test or Fisher's exact test for categorical variables, as appropriate. Effect size Φ was calculated as the Phi coefficient for 2 × 2 tables, effect size *V* as Cramér's *V* for variables with three or more categories, and effect size *r* as |*Z*|/√*N*. Even among variables with significant group differences, all effect sizes were small. * indicates statistical significance *p* < 0.05.

Abbreviations: APACHE II, Acute Physiology and Chronic Health Evaluation II; APACHE III, Acute Physiology and Chronic Health Evaluation III; BMI, body mass index; SAPS II, Simplified Acute Physiology Score II; SOFA, Sequential Organ Failure Assessment.

The results of the multivariable generalized linear models for ICU length of stay and hospital length of stay are shown in Table 2. In the multivariable analyses, no statistically significant association was detected between psychiatric comorbidity and either ICU length of stay or hospital length of stay. Age at ICU admission, ICU admission category, BMI, and APACHE III scores were significantly associated with both outcomes. Compared with emergency surgical admissions, non‐surgical admissions were associated with significantly longer ICU and hospital stays, whereas elective surgical admissions were associated with significantly shorter stays.

**Table 2:** Multivariable generalized linear models for intensive care unit (ICU) length of stay and hospital length of stay.

Variable	Adjusted ratio for ICU length of stay, Exp(*β*) (95% CI)	*p* value	Adjusted ratio for hospital length of stay, Exp(*β*) (95% CI)	*p* value
Psychiatric comorbidity (yes vs. no)	0.91 (0.73–1.13)	0.388	0.95 (0.80–1.12)	0.529
Age at ICU admission (per 1‐year increase)	0.98 (0.98–0.98)	<0.001*	0.99 (0.99–0.99)	<0.001*
Male (vs. female)	0.98 (0.90–1.06)	0.548	1.01 (0.95–1.07)	0.762
ICU admission category				
Non‐surgical (vs. emergency surgery)	1.59 (1.36–1.86)	<0.001*	1.19 (1.06–1.34)	0.005*
Elective surgery (vs. emergency surgery)	0.40 (0.36–0.45)	<0.001*	0.88 (0.81–0.97)	0.007*
BMI (per 1 kg/m^2^ increase)	1.01 (1.01–1.02)	0.003*	0.99 (0.98–1.00)	0.006*
APACHE III score (per 1‐point increase)	1.04 (1.03–1.04)	<0.001*	1.01 (1.01–1.02)	<0.001*

*Note*: The lengths of ICU and hospital stays were analyzed using generalized linear models with a gamma distribution and log link. Exp(*β*) represents the adjusted ratio of length of stay; values greater than 1 indicate a longer stay, whereas values less than 1 indicate a shorter stay. The reference categories were no psychiatric comorbidity, female sex, and emergency surgery. In the multivariable generalized linear models with gamma distribution and log link, psychiatric comorbidity was not significantly associated with either ICU length of stay or hospital length of stay. In contrast, age at ICU admission, ICU admission category, BMI, and APACHE III scores were significantly associated with both outcome variables. According to the omnibus test, the overall model was significant for both outcome variables. * indicates statistical significance *p* < 0.05.

Abbreviations: APACHE III, Acute Physiology and Chronic Health Evaluation III; BMI, body mass index; CI, confidence interval.

In‐hospital death occurred in two patients (2.7%) with psychiatric comorbidity and 103 patients (4.9%) without psychiatric comorbidity. The results of the multivariable logistic regression analysis of in‐hospital mortality are shown in Table 3. No statistically significant association was detected between psychiatric comorbidity and in‐hospital mortality. The ICU admission category was significantly associated with in‐hospital mortality. Compared with emergency surgery, elective surgery was associated with a significantly lower risk of death, whereas non‐surgical admission was associated with a significantly higher risk. The APACHE III score was also significantly associated with in‐hospital mortality, with higher scores indicating higher mortality risk.

**Table 3:** Multivariable logistic regression analysis of in‐hospital mortality.

Variable	aOR (95% CI)	*p* value
Psychiatric comorbidity (yes vs. no)	0.59 (0.13–2.83)	0.514
Age at ICU admission (per 1‐year increase)	1.00 (0.98–1.02)	0.953
Male (vs. female)	1.08 (0.66–1.76)	0.768
ICU admission category		<0.001*
Non‐surgical (vs. emergency surgery)	2.32 (1.24–4.33)	0.009*
Elective surgery (vs. emergency surgery)	0.50 (0.26–0.97)	0.040*
BMI (per 1 kg/m^2^ increase)	1.00 (0.95–1.06)	0.899
APACHE III score (per 1‐point increase)	1.06 (1.05–1.07)	<0.001*

*Note*: All models were adjusted for age, sex, ICU admission category, BMI, and APACHE III scores. The reference categories for the categorical variables were no psychiatric comorbidity, male sex, and emergency surgery. In the multivariate logistic regression analysis of in‐hospital mortality, psychiatric comorbidity was not significantly associated with in‐hospital mortality. In contrast, the APACHE III score was significantly associated with in‐hospital mortality. ICU admission category was also significantly associated with mortality risk: compared with emergency surgery, elective surgery was associated with a significantly lower risk of mortality, whereas non‐surgical admission was associated with a significantly higher risk of mortality. * indicates statistical significance *p* < 0.05.

Abbreviations: aOR, adjusted odds ratio; APACHE III: Acute Physiology and Chronic Health Evaluation III; BMI, body mass index; CI, confidence interval.

## DISCUSSION

This study examined the association between psychiatric comorbidity and clinical outcomes among patients admitted to the ICU. After adjustment for disease severity and patient characteristics, psychiatric comorbidity was not significantly associated with ICU length of stay, hospital length of stay, or in‐hospital mortality. These findings suggest that it is not independently associated with short‐term clinical outcomes in this patient population in the ICU. Furthermore, our findings add evidence concerning Japanese ICU patients to the limited literature on the association between psychiatric comorbidity and short‐term clinical outcomes among critically ill patients.

The prevalence of psychiatric comorbidity in this study was 3.4%, which is lower than the 8.1% reported in ICU populations.^1^ This discrepancy may partly reflect differences in the study population, particularly the absence of patients admitted because of suicide attempts or self‐harm. It may also reflect differences in the ascertainment of psychiatric comorbidity, because our definition required ongoing treatment before hospitalization and therefore did not count ICD‐10 F2–F4 diagnoses without ongoing treatment, whereas Flaws et al. used routinely recorded ICD‐10 diagnostic information. Patients with psychiatric comorbidity were more likely to receive psychiatric consultation during the ICU stay and to use psychotropic medications. Proactive consultation‐liaison psychiatric interventions may reduce hospital length of stay.^15^ Moreover, mental disorders have been associated with adverse clinical outcomes in patients with various physical diseases,^16^ whereas effective treatment of depression may improve outcomes of certain comorbid physical conditions.^17^ Therefore, the higher frequency of psychiatric consultation and psychotropic medication use in the psychiatric comorbidity group may have favorably influenced the observed outcomes.

Several previous studies have reported prolonged hospital stay among patients with psychiatric comorbidity.^3^, ^6^ In addition, a study of ICU patients for burn injuries reported that those patients with psychiatric disorders exhibited greater burn severity and longer ICU and hospital lengths of stay.^13^ Poor physical conditions and greater surgical burden have been suggested as possible contributing factors. However, these studies included self‐harm‐related admissions, individuals with severe trauma, or cases in which it was unclear whether the admission was related to suicide attempts or self‐harm and therefore differed from the patient population in the current study. In our ICU population, which did not include admissions resulting from suicide attempts or self‐harm, psychiatric comorbidity was not significantly associated with either ICU length of stay or hospital length of stay. These findings suggest that ICU and hospital lengths of stay are driven more by the admission diagnosis and physical illness or trauma severity than by psychiatric comorbidity.

Findings regarding mortality have also been inconsistent, with some studies reporting changes in mortality risk before and after adjustment.^7^ In the present study, which focused on an ICU population that did not include patients admitted because of suicide attempts or self‐harm, psychiatric comorbidity was not significantly associated with in‐hospital mortality. However, because only two patients with psychiatric comorbidity died during hospitalization, the association between psychiatric comorbidity and in‐hospital mortality could not be estimated precisely. Additionally, the low number of mortality events may have limited statistical power and increased the risk of Type II error. Therefore, the absence of a statistically significant association should not be interpreted as evidence of equivalent mortality risk between patients with and without psychiatric comorbidity, and the findings should be interpreted cautiously.

This study has several limitations. First, although no significant associations were found between psychiatric comorbidity and ICU length of stay, hospital length of stay, or in‐hospital mortality, the number of patients with psychiatric comorbidity was limited, and small effects may have gone undetected. Second, psychiatric comorbidity was defined as documented ongoing treatment before the index hospitalization for disorders classified as ICD‐10 F2–F4. Untreated or undocumented psychiatric disorders may therefore have been misclassified. Accordingly, our findings should be interpreted as primarily applying to patients with documented and treated ICD‐10 F2–F4 disorders. Third, the severity and duration of psychiatric disorders, details of treatment before ICU admission, and the content, frequency, and continuity of psychiatric interventions were not sufficiently assessed. Fourth, although illness severity was adjusted for using the APACHE III score, residual confounding related to the primary ICU diagnosis and diagnosis‐specific clinical characteristics cannot be excluded. Finally, because this was a retrospective single‐center study, the findings should be generalized cautiously to other institutions or healthcare settings.

No statistically significant association was detected between psychiatric comorbidity and any of the short‐term clinical outcomes examined in this study. However, the association with in‐hospital mortality remains uncertain because of the small number of deaths, particularly in the psychiatric comorbidity group. Future multicenter studies with larger sample sizes are needed to further examine the associations of psychiatric comorbidity with ICU and hospital length of stay and in‐hospital mortality.

## DECLARATION OF GENERATIVE AI AND AI‐ASSISTED TECHNOLOGIES IN THE MANUSCRIPT PREPARATION PROCESS

During the preparation of this work, the authors used ChatGPT (OpenAI) in order to obtain assistance with English translation and language editing. After using this tool/service, the authors reviewed and edited the content as needed and take full responsibility for the content of the published article.

## AUTHOR CONTRIBUTIONS

**Toshinori Nakamura**: Conceptualization; methodology; data curation; formal analysis; writing—original draft; writing—review and editing. **Takumi Momma**: Data curation; writing—original draft; writing—review and editing. **Tomonari Yoshizawa**: Formal analysis; writing—review and editing. **Yusuke Arai**: Supervision; writing—review and editing. **Daimei Sasayama**: Supervision; writing—review and editing. **Atsuyoshi Mita**: Supervision; writing—review and editing. **Hiroshi Imamura**: Supervision; writing—review and editing. **Ryota Hashimoto**: Methodology; supervision; writing—review and editing. **Shinsuke Washizuka**: Supervision; writing—review and editing. All authors have read and approved the final version of the manuscript.

## CONFLICT OF INTEREST STATEMENT

The authors declare no conflicts of interest.

## ETHICS APPROVAL STATEMENT

This study was approved by the Medical Ethics Committee of Shinshu University School of Medicine (Study No. 5871).

## PATIENT CONSENT STATEMENT

Because of its retrospective observational design, the requirement for written informed consent was waived. Nonetheless, information about the study, including the purpose, study period, eligible participants, use of medical records, and protection of personal information, was disclosed on the institutional website. Participants were also informed of their right to opt out from participation without any disadvantages.

## CLINICAL TRIAL REGISTRATION

N/A.

## Supporting information

Supplementary Table 1. Assessment of multicollinearity among independent variables in the model that included all severity indices. The APACHE II, APACHE III, SAPS II, and SOFA were examined as severity indices; however, these measures were highly correlated both conceptually and statistically, and multicollinearity was observed when they were included simultaneously in the model. These findings indicate that including multiple severity indices in the same model is statistically inappropriate. Therefore, in the present study, the APACHE III score was selected as the representative severity index, considering clinical relevance and model stability. APACHE II, Acute Physiology and Chronic Health Evaluation II; APACHE III, Acute Physiology and Chronic Health Evaluation III; BMI, body mass index; ICU, intensive care unit; SAPS II, Simplified Acute Physiology Score II; SOFA, Sequential Organ Failure Assessment; VIF, variance inflation factor.

Supplementary Table 2. Assessment of multicollinearity among the independent variables in the model that included APACHE III as the sole severity index. We calculated VIF values in the linear regression model that included only the APACHE III score. All variables had VIF values below 1.3, indicating no evidence of multicollinearity. APACHE III, Acute Physiology and Chronic Health Evaluation III; BMI, body mass index; ICU, intensive care unit; VIF, variance inflation factor.

## Data Availability

The datasets generated and/or analyzed during the current study are not publicly available because the ethical approval for this study did not include public data sharing and because of the need to protect patient privacy.
